# Deep brain stimulation for movement disorder treatment: exploring frequency-dependent efficacy in a computational network model

**DOI:** 10.1007/s00422-021-00909-2

**Published:** 2021-12-11

**Authors:** Konstantinos Spiliotis, Jens Starke, Denise Franz, Angelika Richter, Rüdiger Köhling

**Affiliations:** 1 Institute of Mathematics, University of Rostock, 18057 Rostock, Germany; 2 Oscar-Langendorff-Institute of Physiology, Rostock University Medical Center, Rostock, Germany; 3 Institute of Pharmacology, Pharmacy and Toxicology, Faculty of Veterinary Medicine, University of Leipzig, Leipzig, Germany

**Keywords:** Mathematical modelling, Neuronal network, Basal ganglia, Movement disorders, Deep brain stimulation (DBS), Synchronization, Macroscopic properties

## Abstract

A large-scale computational model of the basal ganglia network and thalamus is proposed to describe movement disorders and treatment effects of deep brain stimulation (DBS). The model of this complex network considers three areas of the basal ganglia region: the subthalamic nucleus (STN) as target area of DBS, the globus pallidus, both pars externa and pars interna (GPe-GPi), and the thalamus. Parkinsonian conditions are simulated by assuming reduced dopaminergic input and corresponding pronounced inhibitory or disinhibited projections to GPe and GPi. Macroscopic quantities are derived which correlate closely to thalamic responses and hence motor programme fidelity. It can be demonstrated that depending on different levels of striatal projections to the GPe and GPi, the dynamics of these macroscopic quantities (synchronisation index, mean synaptic activity and response efficacy) switch from normal to Parkinsonian conditions. Simulating DBS of the STN affects the dynamics of the entire network, increasing the thalamic activity to levels close to normal, while differing from both normal and Parkinsonian dynamics. Using the mentioned macroscopic quantities, the model proposes optimal DBS frequency ranges above 130 Hz.

## Introduction

### Basal ganglia connectivity

**Fig. 1:**
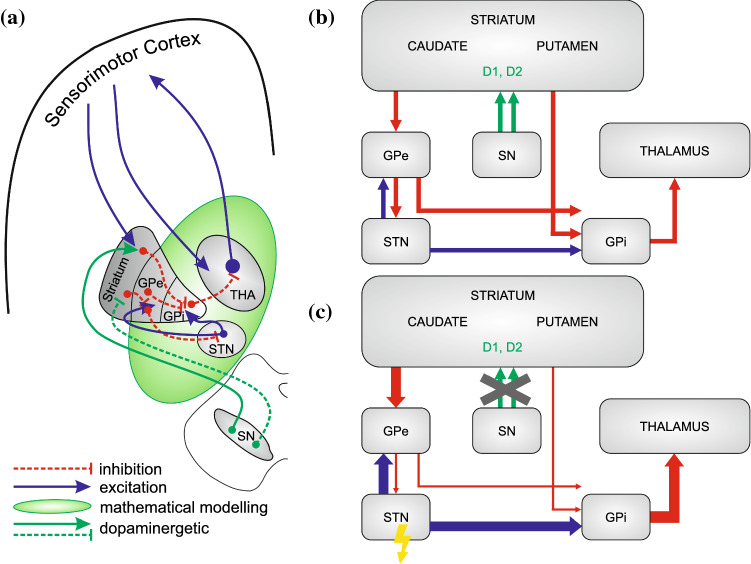
Representation of the basal ganglia and thalamus network and the relevant connections in their approximate anatomical positions **a**, as reduced functional scheme in healthy conditions **b** and under Parkinsonian conditions **c**, where the crossed out green arrows denote the reduced dopaminergic input from the substantia nigra. The striatum is functionally separated into 2 areas: one with predominantly D1-receptors, and the other predominantly expressing D2 receptors. The sensorimotor cortex projects to the thalamus and to the striatum as input region of the basal ganglia. The basal ganglia network consists of the striatum, the globus pallidus, external and internal parts (GPe and GPi, respectively), the subthalamic nucleus (STN), substantia nigra (considering pars compacta here only, SN). The green area in (**a**) marks the part of the network which has been modelled mathematically. Blue lines with arrows depict excitatory connections, while red lines inhibitory ones. Green arrows depict dopaminergic projections from the SN, which will activate the striatum in the direct pathway via D1 receptors, and inhibit the striatum in the indirect pathway via D2 receptors. In (**b**) and (**c**), bold lines depict increases in corresponding synaptic projections and thin lines reductions, respectively. The thunderbolt arrow indicates the target of deep brain stimulation (DBS) in the STN. We hypothesize that under DBS conditions, the increased excitation (bold blue projection from STN to GPi) will be reduced, and the disinhibition (thin red projection from GPe to Gpi) will be normalized

Parkinson’s disease (PD) and dystonia, including different types, belong to the most common movement disorders and hence pose a considerable health burden (Chesselet and Delfs 1996; Defazio 2010; de Lau and Breteler 2006). It is generally accepted that they arise from a dysfunction of the basal ganglia (BG), shown as simplified circuitry in Fig. 1 and result in hypokinetic or hyperkinetic symptoms, depending on which part of the circuitry is affected. In this circuitry, cortical glutamatergic projections are thought to activate GABAergic medium spiny neurons (MSN) and interneurons of corpus striatum (striatum in Fig. 1). From the medium spiny, inhibitory neurons, the so-called direct pathway, project to globus pallidus pars interna (GPi). The inhibition of GPi leads to activation of thalamus (THA) (via disinhibition); one can thus speculate that the thalamus faithfully responds to initial cortical signals (to initiate movement). In the so-called indirect pathway, the activation of striatum inhibits the globus pallidus pars externa (GPe) which projects to the subthalamic nucleus (STN), enhancing its activity. An increased STN activation, in turn, will lead to an increment of GPi activity, resulting in an inhibition of the thalamus (and hence reduction of locomotive activity Calabresi et al. 2014), see also Fig. 1. Parkinson’s disease constitutes a paradigmatic hypokinetic syndrome, which results from the degeneration of the substantia nigra (SN in Fig. 1). It is characterised by rigidity, tremor and hypokinesia, i.e. the inability to start movements fluently (DeLong 1990; Maiti et al. 2017). Looking at the circuitry, the motor symptoms can well be explained by a loss of both activating and inhibitory projections of the SN to the striatum, leading to disinhibition in the so-called indirect (striatum–GPe–STN–GPi–THA) and over-activation in the so-called direct pathways (striatum–GPi–THA) (Calabresi et al. 2014). Hyperkinetic syndromes, characterised by involuntary movements or muscle contractions, in turn, are generally thought to originate from functional or structural damage or degeneration of striatum (dystonia or choreatic syndromes) and of STN; ballistic syndromes. Again, the simplified circuitry depicted in Fig. 1 can serve to explain the functional outcome of e.g. striatal over-activation speculated to result in a shift of balance toward the so-called direct pathway in dystonias (Wichmann and Dostrovsky 2011).

### Role of activity patterns

While the simplified circuitry suggests an explanation for the emergence of hyper- or hypokinetic syndromes, in fact it does not take into consideration the patterning of activity although information in the nervous system is actually conveyed by spatio-temporal activity patterns. Pattern propagation, however, will typically depend on nonlinear couplings between the interacting compartments of the system (Bevan et al. 2002). Hence, one cannot assume that inhibitory or excitatory activity will straight-forwardly propagate across the network. Importantly, both in PD and in dystonia, changes in oscillatory activity patterns in the basal ganglia and the cortex seem to be markers of the diseases (Eusebio and Brown 2007). Thus, in PD, synchronised beta-band activity in both cortex and STN seems to be associated with hypokinesia (Crowell et al. 2012; Kühn et al. 2008). This prominent beta-band is speculated to be caused by the synchronised network activity between the different basal ganglia nuclei (Schmidt et al. 2013). Indeed, a shift of network behaviour from autonomous oscillations of STN and GPe neurons, to synchronous abnormal low-frequency bursting, is observed (Bevan et al. 2002). These findings provide further support for the view that the basal ganglia use both the pattern and the rate of neuronal activity to encode information. Synchronous abnormal patterns of activity of this local circuit should also be reflected in similar changes regarding to the activity of the basal ganglia output and the thalamic activity.

### Deep brain stimulation

Deep brain stimulation (DBS) has been deemed to be the most important innovation in movement disorder therapy and has revolutionised treatment for PD, dystonia and essential tremor patients, first having been approved for the latter condition by the FDA (Food and Drug Administration, USA) in 1997 (Krack et al. 2019). Moreover, DBS is currently being introduced also for the therapy of mental disorders such as depression and obsessive-compulsive disorder (Holtzheimer and Mayberg 2011). DBS improves levodopa-related motor complications in PD and often, motor symptoms in dystonia (Deuschl et al. 2006; Vidailhet et al. 2013). For PD, we know that the efficiency of the treatment depends strongly on the frequency of the stimulus; high-frequency stimulation (HFS), $f>90$ Hz (McConnell et al. 2012) improves motor symptoms, while low frequencies are ineffective or worsen the motor disorders (Koeglsperger et al. 2019). Unfortunately, HFS may worsen frontal functions such as verbal fluency. In contrast to motor functions, very low frequency (10Hz) on STN DBS has significantly better results in verbal fluency (Wojtecki et al. 2006). Until now, the main mechanism of DBS remains elusive (Krack et al. 2019; Ashkan et al. 2017; Udupa and Chen 2015). The main hypotheses put forward so far are (Rubin and Terman 2004; Guo et al. 2008) (a) an inactivation of the target nuclei, (b) changes in transmitter release, (c) neuroprotective and electrostatic effects (i.e. structural plasticity), and network effects resulting in firing pattern alterations. In addition, DBS does not cause a total silencing of neuronal activity of the target nuclei, but rather complex responses. These include sequences of prolonged activation or activity reduction (Luo and Kiss 2016), possibly because of a dissociation between somatal and axonal activation (Holsheimer et al. 2000). One of the most attractive explanations at the moment is the disruption of hypersynchronised oscillations (Kühn et al. 2008). Indeed, recordings from animal models suggest that DBS in the STN results in more periodic and regular firing at higher frequencies in the thalamus (Xu et al. 2008). Thalamic neurons receive inhibitory signals from GPi, an output nucleus of the basal ganglia which provides inhibitory input to the thalamus. Thus GPi activity affects its thalamic targets, generating a possible pathway for STN-DBS, to modify basal ganglia–thalamocortical activity. As a consequence, STN-DBS alters Parkinsonian GPi activity which might improve thalamo-cortical fidelity (Guo et al. 2008; So et al. 2012; Santaniello et al. 2015).

### Predicting network dynamics under DBS using computational approaches

From a computational perspective, one of the main obstacles to explain or predict DBS effects on network activity is the lack of a coherent framework which could bridge the different scales (Deco et al. 2008; Siettos and Starke 2016) of network models ranging from microscopic (cellular activity) to macroscopic (symptom) (Pavlides et al. 2012, 2015). An intermediate level, the mesoscopic level, is related to the dynamics of specific networks of neurons in different nuclei of the basal ganglia. These mesoscopic networks constitute the bridge between micro- and macroscales. Examples of these network dynamics and variations of activity pattern are confirmed in a number of animal model studies in Parkinson models. For instance, DBS in the STN leaves firing rates unaltered in GPe and increases the firing rate in the thalamus (i.e. the nucleus the GPi projects on). While single unit recordings thus indicate changes in firing *rates* in projection areas of the STN during DBS, one overarching motive of all observations is that DBS effectively changes firing patterns (McConnell et al. 2012; Xu et al. 2008; So et al. 2012, 2017; Dorval et al. 2010) as the essential element of its therapeutic success.

More specifically, in McConnell et al. (2012), DBS leads to reduced low-frequency neuronal oscillations in GPe and SNr, increased neuronal oscillations at the stimulation frequency, and increased phase locking with the stimulus pulses. Moreover, coherence within and across the GPe and SNr during HFS was reduced in the band of pathological low-frequency oscillations and increased in the stimulation frequency band. These findings provide evidence that effective high-frequency DBS suppresses low-frequency network oscillations and entrains neurons in the basal ganglia. Therefore, these results support the hypothesis that the effectiveness of HFS stems from its ability to override pathological firing patterns in the basal ganglia by inducing a new regularized pattern of synchronous neuronal activity.

In this spirit we propose a computational large-scale biophysical model related to the Parkinson disease (PD) and DBS treatment. Based on the work presented in Terman et al. (2002); Rubin and Terman (2004) and using complex network theory (Bassett and Bullmore 2006; Watts and Strogatz 1998; Bullmore and Sporns 2009), three areas of the BG: the globus pallidus (partes externa/interna) (GPe-GPi), the subthalamic nucleus (STN), and the thalamus, are modelled. We show, in accordance with the dopaminergic dysfunction during Parkinson’s disease, that different levels of striatal inhibition to BG areas change the behaviour from “normal” to “Parkinsonian”, i.e. switching from faithful transfer of information through the network to a state of disturbed information transmission. The model can also reproduce the action of DBS in the STN and illustrates how high-frequency stimulation (HFS) influences the whole network with respect to its computational features. Specifically, during DBS conditions, the model reveals a de-synchronisation or declustering of GPe and GPi activity which is projected to the thalamus. Defining and quantifying the response efficacy of thalamic activation during DBS, we deduce ranges of stimulation frequencies optimal for therapeutic success. Our model uses a significantly large number of neurons and connections (approx. two orders of magnitude larger compared to Terman et al. 2002; Rubin and Terman 2004; So et al. 2012) which makes the model more realistic and allows us to describe the behaviour of the neural network macroscopically. Although the detailed description is on the microscale (level of neurons), a macroscopic analysis is of interest, specifically the mean activity of interconnected neurons. The results of the macroscopic analysis suggest that a strong nonlinear response is obtained in the basal ganglia network, similar to resonating mechanical systems e.g. (Asadi et al. 2021; Bureau et al. 2014, 2013; Schilder et al. 2015). The following analysis proposes optimal frequencies above 130Hz, suggesting the investigation of DBS treatment with frequencies beyond 130Hz in animal experiments.

**Fig. 2:**
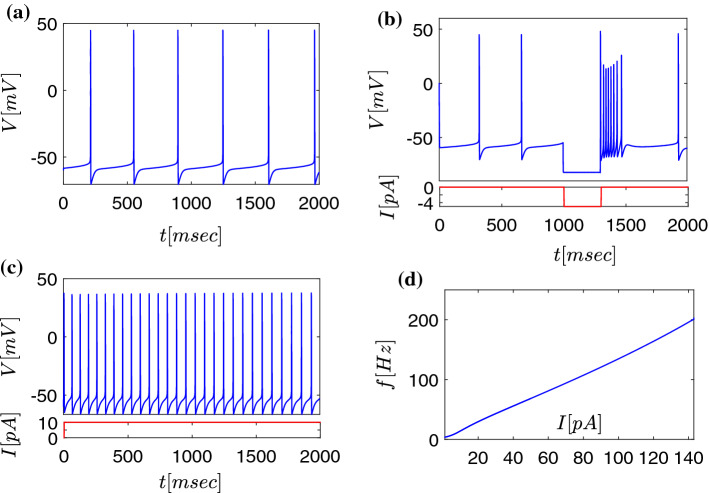
Modelled activity of single STN neurons under different current injection input conditions described by eqs. (1), (2), (3), and resulting current–frequency tuning curve. **a** STN neurons without current injection fire with a frequency around 3 Hz. **b** A negative current injection (current injection depicted in the lower part of the diagram, red curve) applied between t=1.0 and t=1.2 sec results in transient silencing of the neuron and subsequent rebound firing due to $I_{\text {T}}$ (also known as $I_\text {H}$). **c** Injection of 10 pA positive current (current injection depicted in the lower part of the diagram, *red curve*) results in tonic firing activity at 15Hz. **d** Current–frequency tuning curve over the entire range of current injection modelled (0-150 pA)

## Mathematical modelling of basal ganglia and thalamic neurons

Our network model includes in addition to 3 areas of the basal ganglia: the subthalamic nucleus (STN), the globus pallidus internal (GPi) and external (GPe) also a part of the thalamus (THA), see Fig. 1. In this section we formulate the mathematical description for the neurons in each area of the basal ganglia (BG) and the thalamus.

In the model, the STN plays a key role as DBS is applied there to treat Parkinson’s disease. The dynamics of each STN, GPe, and GPi neuron are governed by a Hodgkin–Huxley formalism, and the current balance equation for the membrane potential reads (Terman et al. 2002; Bevan and Wilson 1999; Popovych and Tass 2019):

1 $$\begin{aligned}&C\frac{\mathrm{d}V_i}{\mathrm{d}t} =-I_{\text {LEAK}}-I_{\text {K}}-I_{\text {Na}}-I_{\text {Ca}}-I_{\text {T}}-I_{\text {AHP}} \nonumber \\&\qquad \qquad \quad -I_{\text {syn}}+I_{\text {app}}+I_{\text {DBS}} \end{aligned}$$

2 $$\begin{aligned}&\frac{\mathrm{d}x_i}{\mathrm{d}t} =(x_{\infty }-x_i)/\tau _{x_i} \end{aligned}$$

3 $$\begin{aligned}&\frac{\mathrm{d}[\text {Ca}^{2+}]_i}{\mathrm{d}t} =k_2\left( -I_{\text {Ca}}-I_{\text {T}}-k_{\text {Ca}}[\text {Ca}^{2+}]_i\right) , \end{aligned}$$

where *C* is the membrane capacity, $V_i$ is the membrane potential of the *i*-th neuron, $x_i$ denotes the gating variables *n*, *h*, *r*, and $[\text {Ca}^{2+}]_i$ is the intracellular concentration of calcium. For all basal ganglia areas the currents are described below: The leak currents $I_{\text {LEAK}}=g_{\text {LEAK}}(V_i-E_{\text {LEAK}}),$ the potassium calcium and sodium currents are given by $I_{\text {K}}=g_{\text {K}}n^4(V_i-E_{\text {K}})$, $I_{\text {Ca}}=g_{\text {Ca}}s^2_{\infty }(V_i-E_{\text {Ca}})$ and $I_{\text {Na}}=g_{\text {Na}}m^3_{\infty }h(V_i-E_{\text {Na}})$, while the low-threshold T-type calcium current for STN is $I_{\text {T}}=g_{\text {T}}a^3_{\infty }b^2_{\infty }(V_i-E_{\text {Ca}})$. In the case of GPe, GPi neurons, the low-threshold calcium current has the form $I_{\text {T}}=g_{\text {T}}a^3_{\infty }r(V_i-E_{\text {K}})$, reducing the bursting activity of the GPe relative to STN neurons. The current underlying the after-hyperpolarizing potential has the form $I_{\text {AHP}}=g_{\text {AHP}}([\text {Ca}^{2+}]/(k_1+[\text {Ca}^{2+}])(V_i-E_{\text {K}})$.

The current $I_{\text {DBS}}$ in eq. (1) models the deep brain stimulation of STN neurons and is set to the value 0 in the absence of DBS. The current $I_{\text {app}}$ is applied to the STN, GPe, and GPi, but with different physiological meaning. In the case of STN neurons, $I_{\text {app}}$ simulates the afferent synaptic input from the cortex (Terman et al. 2002), while in the case of GPe and GPi the $I_{\text {app}}$ represents the incoming signal from the striatum with different levels of inhibition of GPe and GPi, respectively.

In eq. (2) the equilibrium state is $$x_{\infty }=x_{\infty }(V_i)=1/(1+e^{-(V_i-\theta _x)/\sigma _x})$$ for $$ x=n,m,h,a,r,s$$, while for the equilibrium state of T-type current the following form is used: $$b_{\infty }(V_i)=1/(1+e^{(r_i-\theta _b)/\sigma _b})-1/(1+e^{-\theta _b/\sigma _b})$$. The voltage-dependent timescale $$\tau _{x}$$ has the form $$\tau _{x}(V_i)=[\tau _{x0}+\tau _{x1}/(1+e^{-(V_i-\theta _{\tau x})/\sigma _{\tau x}})]/ A_x$$, for the STN neurons and $$\tau _{x}(V_i)=\tau $$ for GPe and GPi neurons (Terman et al. 2002).

Figure 2 depicts the dynamics of one (uncoupled) STN neuron, firing at a frequency of 3Hz, while in all simulations we use $I_{\text {app}}=4$pA resulting in an STN activity of 6–7Hz (Bevan and Wilson 1999) (all values of parameters are given in table 1, see also Terman et al. 2002). When a negative current is applied for a short time, the neuron is hyperpolarized accordingly. Due to the presence of hyperpolarization-activated currents (HCN currents), a rebound burst occurs after the current injection.

**Table 1:** Values of parameters that used in section 2 for mathematical modelling

STN	Value	GPe/GPi	Value
$g_{\text {LEAK}}$	2.25 nS/$\mu m^2$	$g_{\text {LEAK}}$	0.1 nS/$\mu m^2$
$g_{\text {K}}$	45.0 nS/$\mu m^2$	$g_{\text {K}}$	30 nS/$\mu m^2$
$g_{\text {Na}}$	37.5 nS $\mu m^2$	$g_{\text {Na}}$	120 nS/$\mu m^2$
$g_{\text {T}}$	0.5 nS/$\mu m^2$	$g_{\text {T}}$	0.5 nS/$\mu m^2$
$g_{\text {Ca}}$	0.5 nS/$\mu m^2$	$g_{\text {Ca}}$	0.15 nS/$\mu m^2$
$g_{\text {AHP}}$	9.0 nS/$\mu m^2$	$g_{\text {AHP}}$	30.0 nS/$\mu m^2$
$E_{\text {LEAK}}$	$-$60.0 mV	$E_{\text {LEAK}}$	$-$55.0 mV
$E_{\text {K}}$	$-$80.0 mV	$E_{\text {K}}$	$-$80.0 mV
$E_{\text {Na}}$	55.0 mV	$E_{\text {Na}}$	55.0 mV
$E_{\text {Ca}}$	140.0 mV	$E_{\text {L}}$	120.0 mV
$\tau _{h1}$	500.0 ms	$\tau _{h1}$	0.27 ms
$\tau _{n1}$	100.0 ms	$\tau _{n1}$	0.27 ms
$\tau _{r1}$	17.5 ms	$\tau _{r1}$	0.05 ms
$\tau _{h0}$	1.0 ms	$\tau _{h0}$	.05 ms
$\tau _{n0}$	1.0 ms	$\tau _{n0}$	1.0 ms
$\tau _{r0}$	40.0 ms	$\tau _{r0}$	30.0 ms
$k_1$	15.0	$k_1$	30.0
$k_{\text {Ca}}$	22.5	$k_{\text {Ca}}$	20
$k_2$	$3.75 \cdot 10^{-5}$ $\text {ms}^{-1}$	$k_2$	$10^{-4}\text {ms}^{-1}$
$\theta _m$	$-$30.0	$\theta _m$	$-$37
$\theta _h$	$-$39.0	$\theta _h$	$-$58
$\theta _n$	$-$32.0	$\theta _n$	$-$50
$\theta _r$	$-$67.0	$\theta _r$	$-$70
$\theta _a$	$-$63.0	$\theta _a$	63.0
$\theta _b$	0.4	$\theta _b$	0.4
$\theta _s$	$-$39.0	$\theta _s$	39.0
$\theta _{\tau h }$	$-$57.0	$\theta _{\tau h }$	$-$40.0
$\theta _{\tau n }$	$-$80.0	$\theta _{\tau n }$	$-$40.0
$\theta _{\tau r }$	68.0	$\theta _{\tau r }$	–
$\sigma _m$	15	$\sigma _m$	10
$\sigma _h$	$-$31	$\sigma _h$	$-$12
$\sigma _n$	8	$\sigma _n$	14
$\sigma _r$	$-$2	$\sigma _r$	$-$2
$\sigma _a$	7.8	$\sigma _a$	2
$\sigma _b$	$-$0.1	$\sigma _b$	–
$\sigma _{\tau h }$	$-$3	$\sigma _{\tau h }$	$-$37
$\sigma _{\tau n }$	$-$26	$\sigma _{\tau n }$	$-$37
$\sigma _{\tau r }$	$-$2.2	$\sigma _{\tau r }$	–
$A_h$	0.75	$A_h$	0.05
$A_n$	0.75	$A_n$	0.05
$A_r$	0.12	$A_r$	2
$\alpha$	5	$\alpha$	2
$\beta$	1	$\beta$	0.08
$\theta _0$	$-$39	$\theta _0$	$-$57
$A_{\text {DBS}}$	200	–	–
$\delta _{\text {DBS}}$	.6 ms	–	–
$T_{\text {DBS}}$	6 ms	–	–

**Fig. 3:**
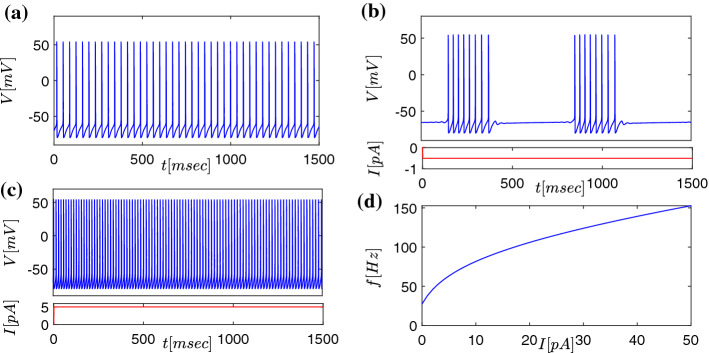
Modelled activity of single GP neurons under different current injection input conditions described by eqs. (1), (2), (3), and resulting current–frequency tuning curves. **a** GP neurons without current injection fire with a frequency around 30 Hz. **b** A constant negative current injection (current injection depicted in the lower part of the diagram, red curve) results in intraburst frequency at 48 Hz. **c** Injection of 5 pA positive current (current injection depicted in the lower part of the diagram, *red curve*) results in tonic firing activity at 63 Hz. **d** Current–frequency tuning curve over the entire range of current injection modelled (0-50 pA)

According to experimental findings (Plenz and Kital 1999; Kita and Kitai 1991), GP neurons show similar ionic currents with respect to the STN cells, yet with different parameters. In the following we explain that the resulting dynamical properties are reproduced by our mathematical model. The main attributes are a spontaneous firing activity at a frequency of $\approx$ 30Hz (Cooper and Stanford 2000; Kita and Kitai 1991), (see also (Terman et al. 2002) and the references there in), and a rebound response to a hyperpolarizing current (Terman et al. 2002; Kita and Kitai 1991; Cooper and Stanford 2000). Simulations of one GPe/GPi neuron are shown in Fig. 3, where the GP neuron fires at a frequency of 30Hz at rest shown in subfigure 3a. Figure 3b depicts the dynamics for small negative $I_{\text {app}}$ resulting in intermittent bursting activity. A small depolarizing current, in turn, results in fast tonic discharges at nearly twice resting discharge frequency 3c. The entire tuning curve depicting responses to different levels of current injections is depicted in 3d.

### Description of the basal ganglia synaptic connectivity

The coupling between the neurons is described by the synaptic current $I_{\text {syn}}$. In the model, GPe and GPi neurons are connected through a Watts and Strogatz (WS) small-world topology (Watts and Strogatz 1998; Bullmore and Sporns 2009; Stam and Reijneveld 2007; Gafarov 2016; De Santos-Sierra et al. 2014; Netoff et al. 2004; Bertalan et al. 2017; Fang et al. 2017), where neurons nearby are densely connected; additionally, a small number of large distance connections exist. Following the experimental findings (Gouty-Colomer et al. 2018) which suggest sparse connections in the STN area, we chose a modified small-world topology with sparse internal connectivity. The detailed description of the network structure is given in sec. 2.3.

The small-world network is considered in the synaptic currents defined by the activation variable *s*, which are given by Laing and Chow (2002), Ermentrout and Terman (2012), Compte et al. (2000):

4 $$\begin{aligned} \frac{\mathrm{d}s_i}{\mathrm{d}t} = \alpha (1-s_i)H(V_i-\theta _{0})-\beta s_i , \end{aligned}$$

where *H*(*V*) is a smooth approximation of a step function, i.e. $H(V)=1/(1+e^{-(V-\theta _x)/\sigma _x}$).

The excitatory and inhibitory synaptic currents for the *i*-th neuron are given, respectively, by

5 $$\begin{aligned} I_{i,\text {Glu}}=g_{\text {Glu}}(V_i-E_{\text {Glu}})\sum _{j}{A_{ij}s_{j}}, \end{aligned}$$

with $$E_{\text {Glu}}=-10mV$$, and

6 $$\begin{aligned} I_{i,\text {GABA}}=g_{\text {GABA}}(V_i-E_{\text {GABA}})\sum _{j}{A_{ij}s_{j}}, \end{aligned}$$

with $E_{\text {GABA}}=-70mV$, where $A_{ij}$ has the value 1 or 0, depending on whether neurons *i* and *j* are connected or not. The summation is taken over all presynaptic neurons.

In the case of STN neurons the $I_{\text {syn}}$ current is given by the summation $I_{\text {syn}}=I_{\text {STST}}+I_{\text {GPST}}$ and indicates the internal excitation between the STN neurons and the incoming inhibition from the GPe neurons, respectively. The excitatory glutaminergic connections within the STN are expressed by $I_{\text {STST}}$ which follows eq. (5), while the inhibitory current $I_{\text {GPST}}$ is given by eq. (6) and expresses the inhibition from the GPe area.

The synaptic current $I_{\text {syn}}$ for the GPe region is defined by $I_{\text {syn}}=I_{\text {GPeGPe}}+I_{\text {STGPe}}$, where the first term $I_{\text {GPeGPe}}$ express the intra-layer inhibitory interaction of GPe neurons (i.e. follows eq. (6)), while $I_{\text {STGPe}}$ describes excitation from STN neurons. For the GPi region the current $I_{\text {syn}}$ is given by $I_{\text {syn}}=I_{\text {GPiGPi}}+I_{\text {GPeGPi}}+I_{\text {STGPi}}$, where the first two terms $I_{\text {GPiGPi}}$ and $I_{\text {GPeGPi}}$ are inhibitory connections, connections from GPi to itself and from GPe to GPi, respectively, while $I_{\text {STGPi}}$ describes excitations from STN neurons. The values of the parameters are given in table 1, see also tables 1, 2 of Terman et al. (2002).

### Modelling and simulations of neurons in the thalamus

**Fig. 4:**
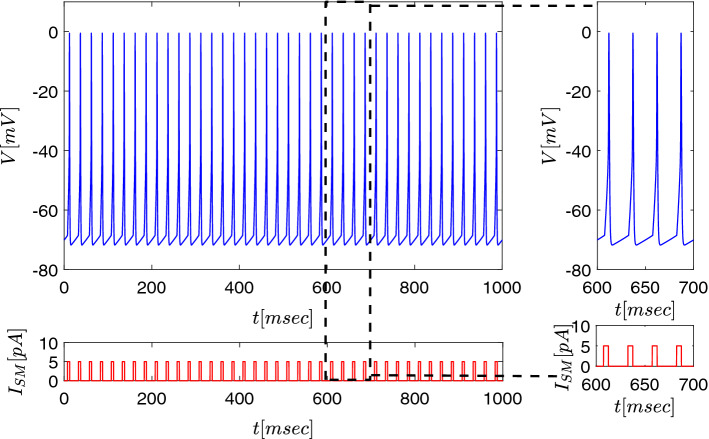
Firing of a single thalamic neuron receiving periodic sensorimotor cortex input current $I_{SM}$. Cortical input was simulated by periodic 5 pA, 5 ms current injections (*red curve*), and corresponding membrane potential changes were calculated (*blue curve*). Isolated thalamic neurons faithfully follow the external periodic stimulus delivered at 40 Hz in this example (see also inset)

Modelling the basal ganglia network, the crucial behaviour of the model is the response of thalamic neurons to synaptic input from GPi neurons. The following section hence addresses this question. Here, a movement programme-associated sensory-motor cortex input to the thalamus is simulated by a repetitive periodic synaptic activation. This is modelled by 5 pA, 5 ms current injections, at 40 Hz (as depicted in Fig. 4), of the form Rubin and Terman (2004): $I_{\text {SM}}=A_{\text {SM}}H(\sin (2\pi t/T_{\text {SM}})\cdot (1-H(\sin (2\pi (t+\delta _{\text {SM}})/T_{\text {SM}}))$, where *H* is the Heaviside function. The GPi neuronal input (resulting, in turn, from basal ganglia network activity) and the sensory-motor cortex input, determine the thalamic firing activity.

The mathematical description of thalamic neurons is given in the following equation

7 $$\begin{aligned} C\frac{\mathrm{d}V_i}{\mathrm{d}t}=-I_{\text {LEAK}}-I_{\text {K}}-I_{\text {Na}}-I_{\text {T}}-I_{\text {GPTH}}+I_{\text {SM}} , \end{aligned}$$

where *C* is the membrane capacity and $V_i$ is the membrane potential of the *i*-th neuron. The leak current has the form $I_{\text {LEAK}}=g_{\text {LEAK}}(V_i-E_{\text {LEAK}}),$ the other ionic currents, i.e. potassium and sodium, are given by $I_{\text {K}}=g_{\text {K}}(0.75(1-h))^4(V_i-E_{\text {K}})$ and $I_{\text {Na}}=g_{\text {Na}}m^3_{\infty }h(V_i-E_{\text {Na}})$, while the low-threshold T-type calcium current is described by $I_{\text {T}}=g_{\text {T}}p^2_{\infty }r(V_i-E_{\text {T}})$. The gating variables *h*, *r* follow the differential equation with first- order kinetics as in eq. (2)

8 $$\begin{aligned} \frac{\mathrm{d}x}{\mathrm{d}t}=(x_{\infty }-x)/\tau _{x} . \end{aligned}$$

The equilibrium function has the form $x_{\infty }(V_i)=1/(1+e^{-(V_i-\theta _x)/\sigma _x})$ for $x=r,h,m,p$, and the voltage-dependent timescale for the gating variable *r* is $\tau _{r}=28+(1+e^{-(V_i+25)/10.5})$, while for *h* is defined as $\tau _{h}=1/(a_h+b_h)$ with $a_h=0.128e^{-(V_i+46)/18}$ and $b_{h}=4/(1+e^{-(V_i+23)/5})$. The current $I_{\text {GPTH}}$ represents the inhibition of the thalamus by the GPi and has the form of eq. (6). For a detailed description of thalamic neurons and for the arithmetic values of parameters, see Rubin and Terman (2004) and table 2.

**Table 2:** The values of parameters that used for THA are given in the next table

THA	Value
$g_{\text {LEAK}}$	0.05 nS/$\mu m^2$
$g_{\text {K}}$	5 nS/$\mu m^2$
$g_{\text {Na}}$	3 nS $\mu m^2$
$g_{\text {T}}$	5 nS/$\mu m^2$
$g_{\text {Ca}}$	0.5 nS/$\mu m^2$
$E_{\text {L}}$	$-$70.0 mV
$E_{\text {K}}$	$-$90.0 mV
$E_{\text {Na}}$	50.0 mV
$E_{\text {Ca}}$	140.0 mV
$E_{\text {T}}$	0 mV
$\tau _{h1}$	500.0 ms
$\tau _{n1}$	100.0 ms
$\tau _{r1}$	17.5 ms
$\tau _{h0}$	1.0 ms
$\tau _{r0}$	40.0 ms
$k_1$	15.0
$\theta _h$	$-$41.0
$\theta _r$	$-$84.0
$\theta _m$	$-$37.0
$\theta _p$	$-$60.0
$\sigma _h$	4
$\sigma _r$	4
$\sigma _m$	7
$\sigma _m$	6.2
$A_{\text {SM}}$	5
$\delta _{\text {SM}}$	5 ms
$T_{\text {SM}}$	25 ms

As Fig. 4 shows, within an isolated cortico-thalamic interaction, the thalamus follows cortical input absolutely faithfully. The next important question is how the basal ganglia network input will modify this strong thalamocortical interplay.

### Network structure

**Fig. 5:**
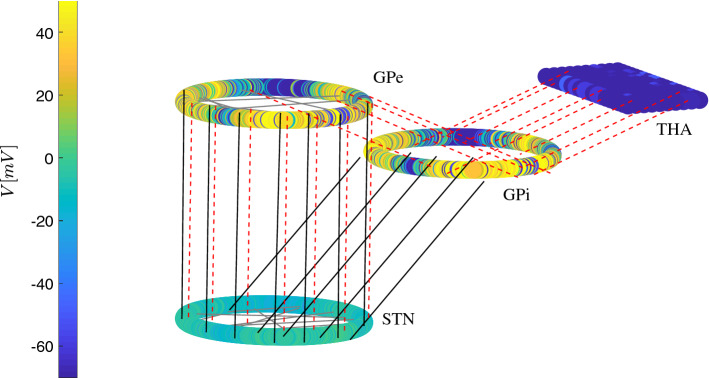
Schematic representation of the basal ganglia network and its activity pattern (voltages of membranes neurons in *colour*). The spatial arrangement is made according to the indices of the neurons, their detailed position in space is not relevant for the model, we use a coordinate system only for visualisation. Black solid lines represent excitatory inputs from STN. Red dotted lines represent inhibitory connections from GPe to STN and GPi, and from GPi to TH, respectively. The GPe and GPi are considered to have a small-world network structure, i.e. also contain sparse long-range connections (*black horizontal lines*), while the STN follows the same structure with reduced number of connections. Each GPe neuron, in turn, is linked to one STN neuron, and vice versa. Furthermore, each STN neuron is connected to one GPi neuron. For the model 500 STN neurons, 500 GPe neurons, 500 GPi neurons, and 200 thalamic neurons were used. From the GPi area, 200 uniformly randomly chosen neurons are connected to thalamic neurons. The ring structures represent relative structural neighbourhood relationships emphasised for GP and STN. For all simulations (healthy, Parkinsonian and DBS) the following synaptic network conductance values were used: $(g_{\mathrm{STST}}, g_{\mathrm{GPST}}, g_{\mathrm{GPeGPe}}, g_{\mathrm{STGPe}}, g_{\mathrm{GPiGPi}}, g_{\mathrm{GPeGPi}}, g_{\mathrm{STGPi}}, g_{\mathrm{GPiTha}}) = (0.5, 4.5, 0.07, 0.56, 0.07, 0.01, 0.2, 0.1)$

For modelling and analysis $N_{\text {STN}}=500$ STN neurons, $N_{\text {GPe}}=500$ GPe neurons, $N_{\text {GPi}}=500$ GPi neurons were used, while the thalamus was represented by $N_{\text {THA}}=200$ neurons. Following (Watts and Strogatz 1998; Bassett and Bullmore 2006; Bullmore and Sporns 2009; Stam and Reijneveld 2007; Spiliotis and Siettos 2011; Shefi et al. 2002), the GPe/GPi layers were modelled as separate small-world networks, i.e. the connections of neurons follow a small-world topology. In such small-world complex networks (Newman 2003), not only does each neuron (node) in the network interact with its *k* nearest neighbours, but there are also a few randomly chosen remote connections (Watts and Strogatz 1998). For the construction of nuclei networks (GPe and GPi) the value $k=20$ was used, while remote connections were created according to Watts and Strogatz (1998) with $p=0.005$. Figure 5 shows a characteristic snapshot of the network. For the STN, we chose a modified approach with reduced internal connectivity, as it is already mentioned in sec. 2.1, matching experimental findings which suggest sparse connections (Gouty-Colomer et al. 2018; Ammari et al. 2010). Specifically, in these experimental findings (Gouty-Colomer et al. 2018), 80% of STN neurons do not have any connections and the remaining 20% of the STN neurons form connections with each other. For these connecting STN neurons, measuring the distance of axonal endings with collaterals (within STN) reveals that roughly 30% of all synapses lie within a 200 $\mu$m radius and another 45% within the 200-400 $\mu$m radius. The other 20% are contacts which occur farther away, i.e. $>500$ $\mu$m (Gouty-Colomer et al. 2018). In this sense, the 20% of neurons, which form STN connections, have both local and remote connections similar to small-world property (i.e. 20% of neurons only showing an average of 25 connections each, see Gouty-Colomer et al. 2018). Finally, the case of denser connectivity between STN neuron is also studied in section 4.5 and the results are shown in Fig. 10.

The coupling between the nuclei is modelled in the following way: Each GPe neuron is connected to one STN neuron and vice versa. Furthermore, each STN neuron projects to one GPi neuron. In addition, the GPe also sends inhibitory signals to the GPi according to a small-world connectivity (one GPe neuron inhibits locally GPi neighbours but also inhibits a few randomly selected remote GPi neurons; in total, one GPe is connected on average with 20 GPi neurons), see Fig. 5. Each thalamic neuron, in turn, is receiving inhibitory inputs from three GPi neurons. Since the main interest of this study is the impact of the basal ganglia output activity on thalamic response to sensorimotor signals (shaping the thalamocortical interplay), this model does not include *intrathalamic* connections. The spatial arrangement in Fig. 5 is made according to the indices of the neurons; their detailed position in space is not relevant for the model (we use a coordinate system only for visualisation). The ring satisfies periodic boundaries conditions.

## Macroscopic description of basal ganglia dynamics

### Synchronisation analysis of basal ganglia dynamics

In the following, a nonlinear dynamical system of the form

9 $$\begin{aligned} \dot{x}=f(x), \quad x\in {\mathbb {R}}^n, \end{aligned}$$

is considered, where *f* is a nonlinear vector field. For a periodic (or oscillatory) solution the property

10 $$\begin{aligned} x(t+T)=x(t), \end{aligned}$$

holds with period *T*, where *x*(*t*) defines a periodic orbit in the phase space. If the periodic orbit shows normal hyperbolicity (Izhikevich and Kuramoto 2004), the system of eqs. (9) can be transformed into an angle or phase equation for the angle $\theta$, i.e.

11 $$\begin{aligned} {\dot{\theta }} = \omega , \end{aligned}$$

where $\omega =2\pi /T$ is the natural frequency of the oscillator.

Coupled oscillators described as system of differential equations (9) are of particular interest in neuroscience. Under certain conditions, identical or nearly identical interacting oscillators converge to a common frequency and synchronise (Strogatz 2001).

The weak coupling of *n* oscillators (Izhikevich and Kuramoto 2004; Kuelbs et al. 2020) is formulated by

12 $$\begin{aligned} \dot{x}_i=f_i(x_i)+\epsilon g_i(x_1,x_2,..,x_n)=f_i(x_i)+\epsilon \sum _{j=1}^{n}g_{ij}(x_i,x_j) , \end{aligned}$$

where $\epsilon \ll 1$ represents the coupling strength. The summation is over the coupling between *j* and *i*, namely over the adjacent of the *i*-th oscillator (connections which project to the *i*-th oscillator). Similarly, there is a transformation which allows to express the dynamics in phase variables, i.e.

13 $$\begin{aligned} {\dot{\theta }}_i&= \omega _i+Q(\theta _i)\epsilon h_i(\theta _1,\theta _2,...,\theta _n) \end{aligned}$$

14 $$\begin{aligned}&= \omega _i+\epsilon \sum _{j=1}^{n}h_{ij}(\theta _i,\theta _j) \end{aligned}$$

where $\theta _i \in [0,2\pi ]$. In the presence of external stimulation (e.g. DBS), eq. (12) can be generalised (Monga et al. 2019; Kuelbs et al. 2020), by including a time-periodic function on the rhs. The weakly coupled system in terms of the phase variable $\theta$, i.e. eq. (14), shows a phase-locked solution if there is a constant integer matrix $K_{n-1,n}$, such that $K\cdot \theta =c$ with $c_k>0$ (Izhikevich and Kuramoto 2004). Furthermore, the coupled oscillators are synchronised (in-phase) when

15 $$\begin{aligned} \theta _1(t) = \theta _2(t)=... = \theta _n(t)=\theta (t) . \end{aligned}$$

Following the approach of Kuramoto (Arenas et al. 2008; Kuramoto 1984b, a; Bertalan et al. 2017) which is applied when oscillators are near a supercritical Andronov–Hopf bifurcation, in case of fully connected network eq. (14), we obtain

16 $$\begin{aligned} {\dot{\theta }}_i = \omega _i+k/N\sum _{j=1}^{N} \sin (\theta _i-\theta _j), \end{aligned}$$

while with arbitrary connectivity i.e. a complex network topology

17 $$\begin{aligned} {\dot{\theta }}_i = \omega _i+k/N\sum _{j=1}^{N}A_{ij}\sin (\theta _i-\theta _j) , \end{aligned}$$

where the coefficient $A_{ij}\in \{0,1\}$ is derived from the network adjacency matrix (Bertalan et al. 2017). The emerging macroscopic dynamics (in terms of phase) can be obtained by taking the mean value of all phase populations (in exponential form, $e^{i\theta }$). This defines the *synchronisation index r*, i.e. (Kuramoto 1984b; Strogatz 2001; Kuelbs et al. 2020)

18 $$\begin{aligned} r(t)=\left| \frac{1}{N}\sum _{k=1}^{N}e^{i\theta _k(t)}\right| \end{aligned}$$

This index is used in the following sections as a measure describing the level of synchronised patterns within the GPi.

The synchronisation index acts as macroscopic variable (order parameter) with range $r\in [0,1]$. In the case of perfect synchronisation, i.e. eq. (15), the index can be written as

19 $$\begin{aligned} r=\left| \frac{1}{N}\sum _{k=1}^{N}e^{i\theta _k}\right| =\frac{1}{N}N \left| e^{i\theta }\right| =1 , \end{aligned}$$

while the case of $r \rightarrow 0$ corresponds to incoherent phase dynamics. The phase $\theta _k(t)$ used in eq. (18) of the *k*-th neuron can be approximated linearly according to the following equation

20 $$\begin{aligned} \theta _k(t)=2\pi \frac{t-t_n}{t_{n+1}-t_n}+2\pi n , \end{aligned}$$

where $t_n$ corresponds to *n*-th firing time of the *k*-th neuron and $t\in [t_n, t_{n+1}]$. Another option to compute the phase without assuming a linear dependence of the angle $\theta$ on time, see eq. (20) is the Hilbert transform, applied by Gabor in Gabor (1946). For a given function $x=x(t)$, the Hilbert transform is defined as

21 $$\begin{aligned} X(t)=\frac{1}{\pi }P\int _{-\infty }^{\infty }\frac{x(\tau )}{t-\tau }d\tau \end{aligned}$$

with *P* denoting the Cauchy principal value. The complex signal $z=z(t)=x(t)+iX(t)=A(t)e^{i\theta (t)}$ is defined in order to extract the phase, where $A, \theta$ is the amplitude and the phase of the complex signal *z*, respectively. The instantaneous phase is defined as:

22 $$\begin{aligned} \theta (t)=\arctan {(X(t)/x(t))} . \end{aligned}$$

The synchronisation index eq. (18) will be computed in the next section to characterise the network activity for the cases of normal, Parkinsonian, and DBS treatment.

**Fig. 6:**
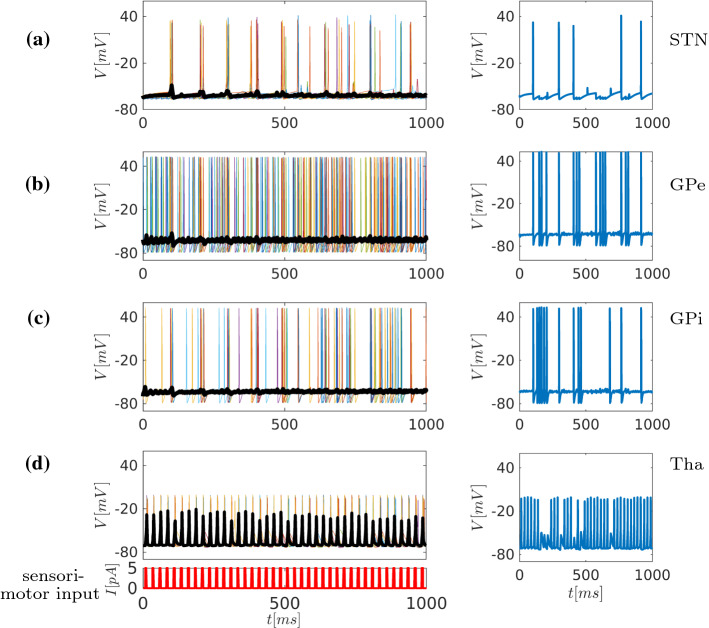
Time series representation of the network dynamics under normal conditions (Fig. 1b). The left column depicts 10 randomly chosen neurons from each area in the network, while the thick black line shows the average membrane potential V of all neurons in the area. The right column depicts time series of single representative neurons in each of the nuclei (STN, GPe, GPi, Tha), as indicated. The STN activity resulting from a continuous current input to STN (simulating afferent synaptic input). At the same time, the thalamus receives periodic sensorimotor input (simulated by rhythmic 5 pA current injections). **a** Under these conditions, the time-dependent activity of all neurons in STN results in nonsynchronised firing at approx. 4-7 Hz. **b** In GPe, as the plot of time-dependent activity of neurons shows, this results in higher-frequency bursting uncorrelated to STN activity. **c** The time-dependent activity in GPi shows a lower-frequency irregular bursting of the neurons. **d** The time-dependent activity plot of the neurons in thalamus demonstrates that the combined input from GPi and sensorimotor drive results in a periodic firing relatively faithfully following the sensorimotor input

**Fig. 7:**
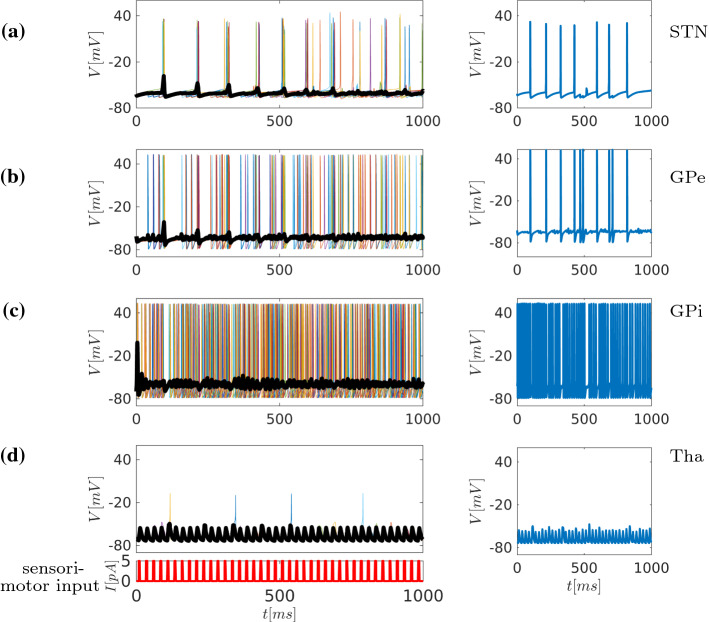
Time series representation of the network dynamics under Parkinsonian conditions (Fig. 1c). The left column depicts 10 randomly chosen neurons from each area in the network, while the thick black line shows the average membrane potential V of all neurons in the area. The right column depicts time series of one representative neuron in each of the nuclei (STN, GPe, GPi, Tha) as indicated. We simulate synaptic input to the STN and thalamic periodic sensorimotor input identical to the normal condition. **a** Under Parkinsonian conditions, the continuous current input to STN (simulating afferent synaptic input) results in an increase in STN activity (raising the frequency from  7 to  11 Hz) at a more synchronised level (see occasional simultaneous firing of one cell in the right column) **b** In GPe, as the plot of time-dependent activity of all neurons shows, this results in a reduction of firing frequency compared to normal conditions, i.e. low-frequency bursting which is more coupled to STN activity (compare in right column). **c** Time-dependent activity in GPi shows bursting at a frequency, 4–5 times higher than under normal conditions, which results in strong inhibition of the thalamus. **d** Indeed, this inhibition is reflected in the time-dependent activity plot of all neurons in thalamus. It demonstrates that the combined input from GPi and sensorimotor drive now results in very sparse firing and essentially a loss of sensorimotor-thalamic coupling

**Fig. 8:**
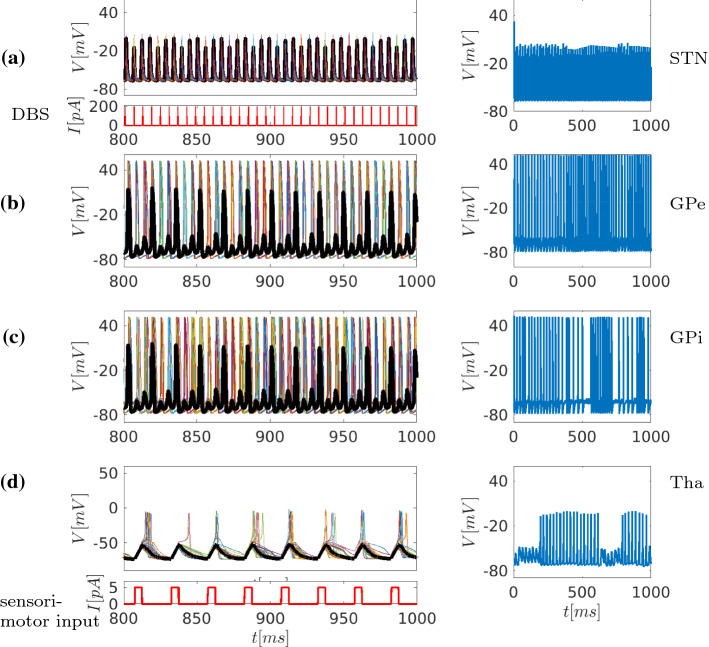
Time series representation of the network dynamics under Parkinsonian conditions (Fig. 1c), but now simulating DBS by high-frequency current injection into STN. The left column depicts randomly chosen neurons from each area in the network, while the thick black line shows the average membrane potential V of all neurons in the area. The right column depicts time series of one representative neuron in each of the nuclei as indicated. For better discrimination in the left column, the last 200 ms is shown. Right columns show activity of representative single neurons for each of the nuclei, as indicated. The thalamus continues to receive periodic sensorimotor input (simulated by rhythmic 5 pA current injections). **a** Under Parkinsonian conditions and DBS simulation, the time-dependent activity of all neurons in STN converts from periodic into tonic high-frequency firing, following the DBS input. **b** In GPe, as the plot of time-dependent activity of all neurons shows, this results in a similarly tonic, regular firing at a slightly lower frequency (elevated compared to normal and even more so compared to Parkinsonian state). **c** In GPi, in turn, the time-dependent activity plot shows that this altered STN and GPe activity is translated into slightly irregular firing at even lower frequency, very close to the normal state. Losing the high-frequency discharges in GPi , the inhibitory drive to the thalamus is reduced, which is reflected also in the reduction of the mean activity index, see also Fig. 12c. **d** This disinhibition is reflected in the time-dependent activity plot of the neurons in thalamus. It demonstrates that the combined input from GPi and sensorimotor drive, together with DBS to STN, now stably restores firing in approximate synchrony to sensorimotor input

**Fig. 9:**
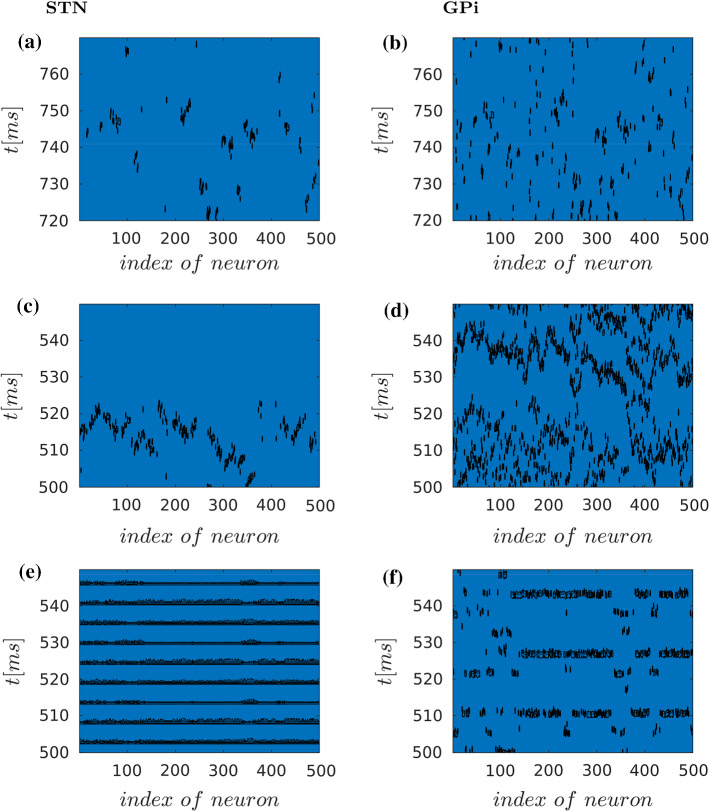
Spatio-temporal activity patterns for STN (*left column*) and GPi (*right column*) under normal (**a** and **b**), Parkinsonian (**c** and **d**) and DBS (**e** and **f**) conditions. Black dots depict activated neurons (i.e. actions potentials defined as transients passing $V=-15$mV to positive values) against time (in ms) and space (i.e. index of neuron of the nuclei, see also Fig. 5)

**Fig. 10:**
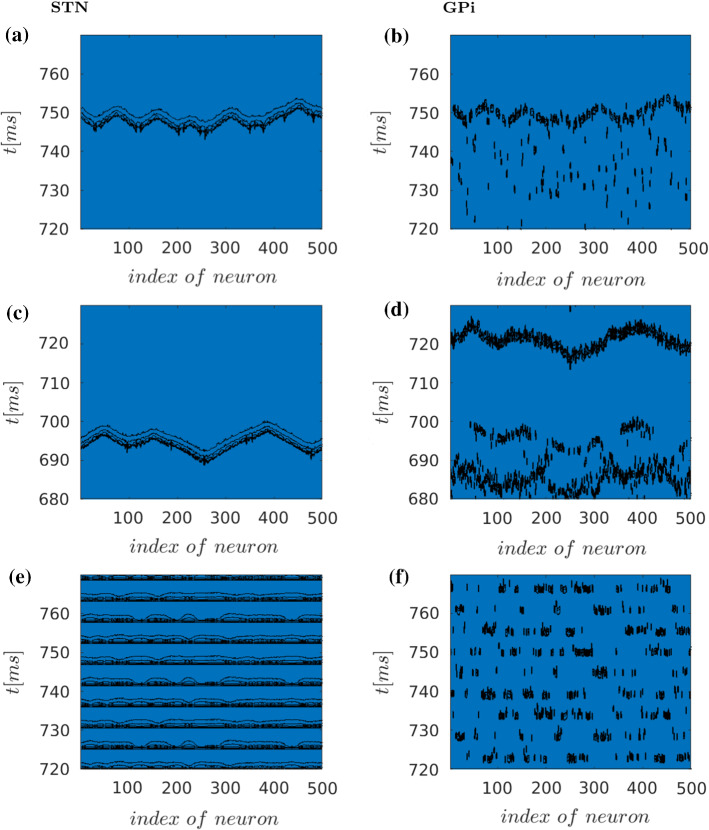
Spatio-temporal activity patterns of basal ganglia network with the assumption of strong connectivity of STN area, for STN (*left column*) and GPi (*right column*) under normal (**a** and **b**), Parkinsonian (**b** and **c**) and DBS (**e** and **f**) conditions. Black dots depict the activated neurons (i.e. actions potentials defined as transients passing $V=-15$mV to positive values) against time (in ms) and space (i.e. index of neuron of the nuclei, see also Fig. 5). The spatio-temporal activity in the regions of STN and GPi strong indicates travelling wave solutions with more variable structures in the normal state

**Fig. 11:**
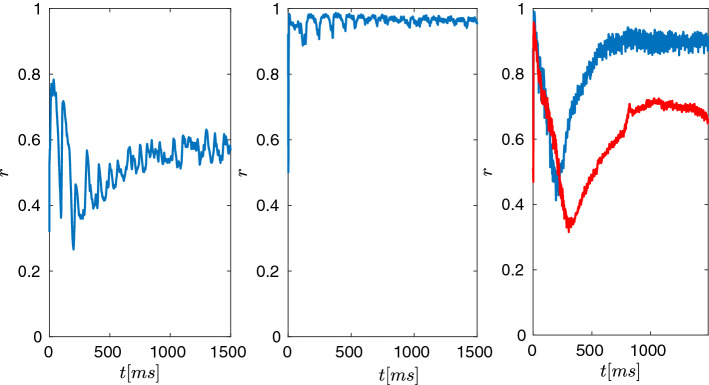
Synchronisation index *r*, defined in eqs. (18, 21) for **a** healthy normal, **b** Parkinsonian state, and **c** DBS. **a** In the healthy state, the synchronisation index shows an oscillatory behaviour at frequency of $\approx$11 Hz with relatively low values of range [0.3, 0.6]. **b** In the Parkinsonian state, in turn, the synchronisation index is high. The index fluctuates irregularly for longer periods during the bursting activity of the GPi neurons. This bursting activity is highly frequent and prolonged, shifting to enhanced $\beta$ power. As a result, the inhibitory drive to the thalamus is strongly increased. **c** Simulating STN-DBS, the synchronisation index for 184Hz (*blue curve*) and 210Hz (*red curve*). For both frequencies, *r* starts out at almost 1 and then wanes down to $\approx$0.4 in periodic, high-frequency dips, with ongoing tonic firing in GPi, where $\beta$ activity is lost. Importantly the higher frequency i.e. 210Hz provides higher de-synchronisation in GPi activity. This also coincides with decreased activity of GPi area, see Fig. 12c

**Fig. 12:**
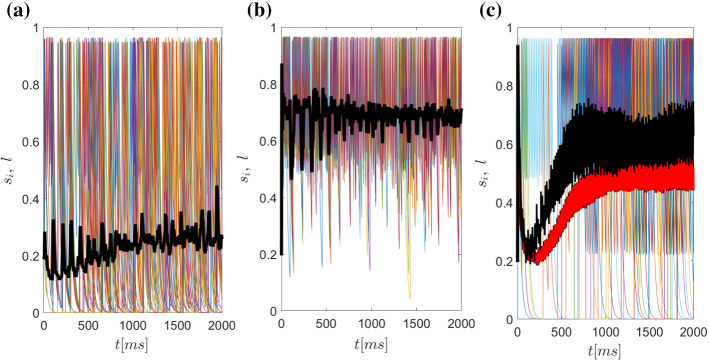
Synaptic activity *l* of GPi projecting to the thalamus over time *t* defined in (23). Both the activities of cells (thin lines, different colours) and the mean of the activity (black and red thick lines) are depicted. **a** Under healthy conditions, the inhibitory input to the thalamus occurs periodically in the low $\beta$ range, and more importantly, in between these inhibitory periods, the degree of synaptic GABAergic activity projecting to the thalamus is low (at $\approx$0.2). **b** Under Parkinsonian conditions, in turn, the situation completely reverses: Presumably due to prolonged burst firing, the mean synaptic activity *l* is generally high ($\approx$0.7) and it drops intermittently during pauses of the GPi bursts. The GPi neural activity is characterised by $\beta$ range. **c** Under STN-DBS for 184 (*black*) and 210 Hz (*red*), the inhibitory drive tonically equilibrates at a mean level of $\approx$ 0.6 for 184Hz; and around 0.5 at 210Hz. In the case of 210Hz (*red colour*) the inhibitory tones is lower than the 184 (*black colour*)

**Fig. 13:**
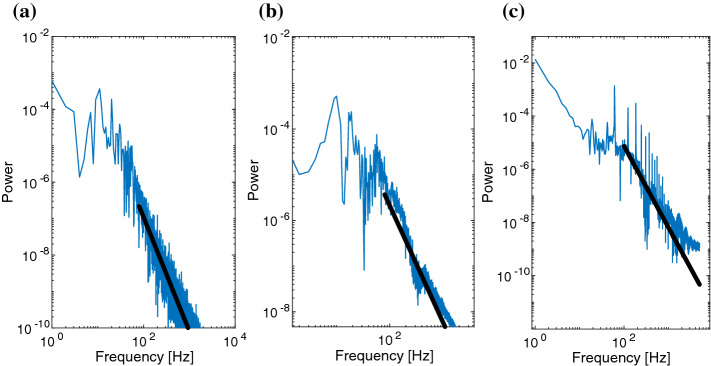
Fourier spectrum of the mean GPi synaptic activity for normal, Parkinsonian, and DBS conditions. **a** Normal state, the power shows 2 peaks at $\approx$7 and 11 Hz representing the mean synaptic variable LFP. Using linear approximation we obtain the slope of the line around −3.6. **b** Fourier spectrum of the mean GPi synaptic activity under Parkinsonian conditions. The power shows strong peaks at 9Hz and a secondary at 18Hz reflecting the synchronisation index fluctuations of shown in Fig. 11b and Fig. 12b. The slope of linear approximation was calculated $\approx$-2.5. **c** Fourier spectrum of the mean synaptic activity projecting from GPi to THA under STN-DBS conditions. The power peaks at $\approx$60 Hz, with additional sharp peaks between $\approx$ 122 and 184 Hz, i.e. corresponding harmonics, reflecting mean synaptic activity Fig. 12c and the synchronisation index fluctuations shown in Fig. 11c. The slope is changed −3.1 close to normal conditions

An alternative macroscopic quantity which can be used to estimate neuronal activity is the *mean synaptic activity index l*. It is defined by

23 $$\begin{aligned} l(t)=\frac{1}{N}\sum _{i=1}^{N}s_i(t) , \end{aligned}$$

where $s_i$ is the synaptic variable as it is defined from eq. (4). Coupling theoretical modelling tasks with experimental data, the synchronisation property in essence reflects local field potentials (LFP). The flow of the extracellular current generating the LFP is represented by the summed up postsynaptic potentials from local cell groups (similar to eq. (23), see Buzsáki 2004; Popovych and Tass 2019; Manos et al. 2018; Popovych and Tass 2018). In the next section this mean synaptic activity will be used to measure the transfer of neuronal activity (current flow information) from the basal ganglia output, i.e. from GPi to thalamus.

## Simulating different functional states of the basal ganglia network

Three cases of dynamic network behaviours were studied. In all cases the thalamus receives a periodic sensorimotor input (simulated by periodic 5 pA current injections Rubin and Terman 2004) which represents the signal for the initiation of movement. In addition, a continuous input current to STN is applied in order to obtain rhythmic activity (simulating afferent synaptic input from cortex Terman et al. 2002). During the first, normal case, see Fig. 1b, basal ganglia network activity allows a relatively faithful response of the thalamic neurons, closely following the sensorimotor cortex input. The second case considered is the Parkinsonian state, depicted schematically in Fig. (1c), with resulting overall increase in inhibitory projections to the thalamus. The third case considers the situation of therapeutic intervention with deep brain stimulation (DBS) to STN, which is simulated as a high-frequency current injection into STN neurons, resulting in switching the entire network dynamics and restoring functional thalamic output close to normal behaviour.

In order to capture the transitions and to measure qualitatively the impact of the network dynamics, we additionally defined macroscopic variables or observables such as the *synchronisation index* and the *average synaptic activity*, focusing on GPi as output region of the basal ganglia network.

All simulations are made with MATLAB 2020a using the solver ode23, a adaptive time step integration Runge–Kutta scheme. The order of the method is three, and the default relative and absolute tolerances in MATLAB $[10^{-6},10^{-6}]$ are used.

### Modelling the normal state

**Fig. 14:**
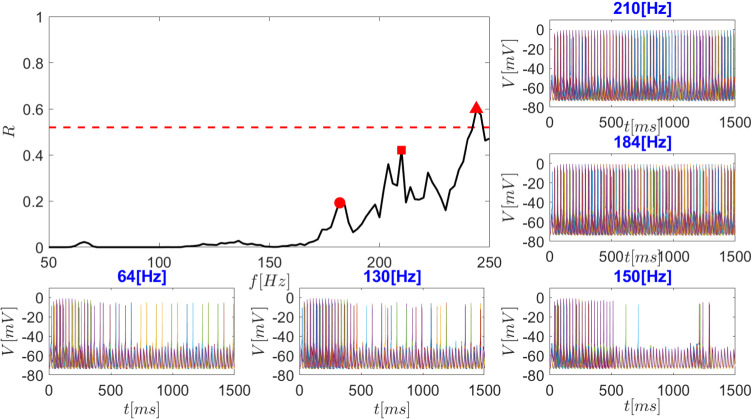
The response efficacy *R* of thalamic neurons changes with DBS frequency *f*. The response efficacy *R* was computed for times $500<t<1500$. The central graph depicts the response efficacy of thalamic neurons to cortical input under DBS. In this context, a value of 1 means that the thalamic neuronal firing is perfectly correlated with the cortical input, a value of 0 that there is no correlation whatsoever. Under normal conditions (dashed line), thalamic neuronal firing shows intermediate correlation to cortical input, with a response efficacy of 0.518 (compare also Fig. 6d), whereas under Parkinsonian conditions, this correlation is almost completely lost, with response efficacy close to 0 (compare Fig. 7d. In our modelling study, using 184 Hz as DBS frequency, the response efficacy is close normal (i.e. 0.22; see first peak of the curve highlighted by filled circle). The insets show the resulting firing behaviour of thalamic neurons in response to cortical input at the different frequencies *f* as indicated. Note that at 150 Hz, the impact of DBS is only transient, lasting only up to 500 ms

Parameters were tuned to simulate normal-healthy conditions as shown in Fig. 1b. Inhibitory signals from the striatum to the GPe are described by the current $I_{\text {app}}=5 \text {pA}$, whereas from striatum to GPi the current has the value $I_{\text {app}}=4$pA. Figure 6 depicts the time-dependent activity of all nuclei under normal conditions. The STN neurons fire irregularly at around 4–7 Hz, i.e. close to values reported in the literature (cf. Bevan and Wilson (1999) Rubin et al. 2012), see Fig. 6a. The GPe activity is plotted in Fig. 6b. It is characterised by high-frequency irregular bursting firing with individual clustering of action potential series (which is not visible on population scale, but only on the level of a single neuron as shown in the right column). Overall, there is little correlation between STN and GPe activity. Similar dynamics appear in GPi, which, in turn, also shows high-frequency firing. Looking more closely, there is, however, an underlying weak rhythmicity at approx. 12 Hz, i.e. in the $\beta$-band, see Fig. 6, and also 12(a). The resulting time-dependent activity plot of all neurons in the thalamus is shown in Fig. 6d, demonstrating that under these conditions, the thalamus faithfully responds to sensorimotor input (Fig. 6e), without however being in a perfect phase-locked state as in the case of single neuron, Fig. 4. Accordingly, the thalamic response efficacy *R* (a macroscopic variable which measures the response of the thalamic neurons to sensorimotor input) is approximately 0.5 under normal conditions. The response efficacy *R* ranges in the interval [0,1], while the value 1 corresponds to an activation of the whole thalamus (exact definition of *R*, see sec. 6 and Fig. 14). In conclusion, the GABAergic synaptic output from the GPi to the thalamus converts thalamic responses (otherwise phase locked to the cortical input) to more loosely firing of the thalamic neurons, without subduing it altogether (thalamic activity $R=0.5$) as in the Parkinsonian state (see Fig. 7).

### Modelling the Parkinsonian state

As outlined above, in Parkinson’s disease, a degeneration of nigrostriatal dopaminergic neurons, accompanied by a reduction in the number of dendritic spines of striatal medium spiny neurons (Gagnon et al. 2017; Fiore et al. 2016), leads to a loss of dopamine in the striatum. The resulting overall reduction of D1/D2 receptor-mediated activity affects the direct/indirect pathway functionality. In the direct pathway, the (D1) receptors malfunction results in disfacilitation of striatal projection neurons and as a consequence a reduced inhibition of the GPi neurons. Thus, the disinhibited GPi increases its neuronal activity, sending higher levels of inhibition to the thalamus.

In the indirect pathway, loss of D2-receptor activation will disinhibit striatal projection neurons. These, in turn, now decrease the activity of the GPe, to which they project. By inhibiting the GPe, the activation of STN is increased, and the overactive STN will enhance the neuronal activity in the GPi which again leads to even more pronounced thalamic inhibition, see Fig. 1c.

Consistent with this concept, the model assumes a decrease in the level of inhibition of GPi neurons $I_{\text {app}}$. This is modelled by increasing depolarising current from 4 to 8 pA (corresponding to disinhibition). At the same time, the model assumes an increase in the level of inhibition of GPe neurons (therefore, the depolarising current is decreased from 5 to 3pA). Figure 7 shows the dynamics of the network under Parkinsonian conditions. The time-dependent activity of all neurons in STN is changed compared to the normal state: Firing becomes more regular and occurs at higher frequency (approx. 11 Hz). For the GPe, Fig. 7b, the consequence is a lower burst frequency, almost following the STN activation (see insets). Figure 7c shows that in GPi, the altered activity is translated to high-frequency bursting, with an underlying enhanced rhythmicity in $\beta$-activity (13-15 Hz) compared to the normal condition, see also Fig. 12b. As a result, inhibition to the thalamus strongly increases, and the thalamus is no longer able to transmit signals in response to sensorimotor input, as its firing becomes very sparse.

### Modelling the effects of DBS

The network structure and the conductances were kept invariant with respect to Parkinsonian conditions. DBS treatment was simulated by a high-frequency current of 184 Hz, applied to all STN neurons (this value resulted after the analysis in section 6 and is suggested as candidate for an optimal DBS frequency in experiments). The high-frequency current is modelled as periodic short pulses of the form (Rubin and Terman 2004)

24 $$\begin{aligned} I_{\text {DBS}}= & {} A_{\text {DBS}}H(\sin (2\pi t/T_{\text {DBS}})\nonumber \\&\cdot (1-H(\sin (2\pi (t+\delta _{\text {DBS}})/T_{\text {DBS}})) . \end{aligned}$$

In all three basal ganglia nuclei and thalamus, STN-DBS induces dramatic alternations in firing dynamics. Regarding the STN, neurons follow the strong DBS signal and fire tonically at stimulation frequency, see Fig. 8a. In GPe, this results in regular bursting of neurons at about 90 Hz, i.e. at  3x higher frequency than normal and  4x higher frequency than under Parkinsonian conditions, respectively. In the GPi, in turn, the increased activity in GPe nearly normalises the firing to slightly irregular firing at  30 Hz (compare insets in this figure and 6) and thus strongly reduces the firing frequency compared to the Parkinsonian state (which ranged around 50–80 Hz) see Fig. 8. In summary, there is an overall loss of synchronisation between STN, GPE, and GPi. Simultaneously the STN and GPe nuclei now fire tonically. With the reduction of the high-frequency tonic firing of the GPi, the thalamus is disinhibited and resumes firing, following the sensorimotor input relatively closely.

### STN-GPi interaction: synchronisation changes and local travelling waves formation

Different spatio-temporal patterns of activation in STN on the one hand, and GPi on the other, can be observed under normal, Parkinsonian, and DBS conditions. Figure 9 depicts the whole neuronal activity of STN and GPi regions in the case of sparse connectivity between STN neurons. In normal case (a) and (b), both areas STN and GPi show irregular patterns. By contrast, in the Parkinsonian case (c) and (d), the regions show higher synchronous activation. Figure  9c shows sparse, but synchronous firing in STN in the Parkinsonian case, with local travelling waves. These waves are restricted to a few neurons, demonstrating clustered organisation of neuronal activity. An interpretation for this is that in the Parkinsonian state, the reduced inhibition from GPe to the STN leads to a higher spatial synchronisation between these nuclei, resulting in a different pattern formation with respect to normal case 9a, b.

Which consequence does this have for the activity in the GPi?

This nucleus receives both input from the STN (indirect pathway) and from the striatum (direct pathway), and hence one would expect two competing activity patterns. Under Parkinsonian conditions, in turn, the inhibitory activity of the direct pathway is reduced. Hence the activity in the GPi is more pronounced, which shows series of quasi-synchronous activations of GPi neurons, with local travelling waves forming small clusters.

Under DBS conditions, the massive synchronisation in the STN (abolishing all travelling waves and imposing a 184 Hz rhythm on nearly all neurons) is mirrored in a closely matching synchronised activity pattern in the GPi, but at lower frequency of around 60Hz, again overriding all other activity. This presumably results in a nearly tonic activation of inhibitory neurons, to which the thalamic neurons desensitise, as will be discussed below.

### Effects of dense STN connectivity

Since the degree of connectivity among STN neurons is still under debate (Amadeus Steiner et al. 2019), in a next step also a higher connectivity was considered. The increment of connectivity, i.e. higher number of connections between STN neurons, leads to different spatio-temporal pattern. Figure 10 shows the normal, Parkinsonian, and DBS states. The qualitative difference here is the strong connectivity within STN. Each neuron now has mutual connections with other neurons, with a mean number of connections equal to 20. The STN structure is described as a small-world topology. Under normal and Parkinsonian conditions, the basal ganglia network depicts strongly correlating travelling waves, which propagate and are more variable in the normal state. The travelling wave represents a propagation of similar activity levels along one direction, i.e. as activity peaks in neighbouring neurons in a co-moving frame, preserving the activity shape. Also with this much higher intra-STN connectivity, the Parkinsonian state is characterised by increased synchronisation in GPi. Under DBS, rhythmic activity of STN in turn is reflected in less synchronisation in GPI. Thus, the model is rather robust with respect to GPi output, within a wide range of STN connectivity.

## Synchronisation and synaptic activity indices characterise transitions of network dynamics

In order to shed light on the transitions between the different states, and to examine the appearance of distinct patterns in our model, we analysed both the level of synchronisation within the GPi (computing the synchronisation index *r*) and the levels of synaptic GABAergic projection activity from the GPi to the thalamus (defining GPi activity index *l*).

The theory of phase synchronisation (Izhikevich and Kuramoto 2004; Ermentrout and Terman 2012; Tass 1999; Pikovsky et al. 2001; Kuramoto 1984b) described in section 3.1, allows us to characterise and analyse different attributes and the dynamics which results from the mathematical model. The main observables are the synchronisation index *r* defined in eq. (18) and the mean GPi synaptic activity index *l* defined in eq. (23) and depicted in Figs. 11 and 12. We further performed a Fourier analysis of the GPi mean synaptic activity index *l*, in order to measure the dominant frequencies (rhythms), and we determined its slope of the power spectrum to estimate the distribution of frequencies in the power spectrum (see Fig. 13).

### Macroscopic dynamics in the normal state

Considering the healthy, normal state, as Fig. 11a shows, synchronisation within the GPi is generally low (around 0.5). The synchronisation index shows oscillatory behaviour (at around 12 Hz). The local maxima of the synchronisation index thus correspond to increased synchronous activation of GPI neurons in the low $\beta$ range. The GPi activity results in an overall periodic, but low-magnitude inhibitory drive to the thalamus, see 12a. Corresponding to this behaviour, the mean synaptic GPi activity phasically oscillates around 0.2 (even if the microscopic behaviour of neurons forms bursting clusters) see, Fig. 12a. This rhythm lies within the lower frequencies (i.e. $\alpha$ and lower $\beta$ band), see Fig. 13a. The spectrum analysis reveals a first peak at 7 Hz and a higher peak at around 11 Hz, i.e. activity in the lower $\beta$ band. This rhythmic peak is centred relatively narrowly around this dominating frequencies, since the slope of linear approximation is relatively steep (around $-3.6$ Hz/sec), meaning that the higher frequencies do not contribute in the magnitude of the power spectrum.

### Macroscopic dynamics in the Parkinsonian state

Under Parkinsonian conditions, the GPi neurons are generally much more active than in the normal state. The synchronisation is very high (close to 1). This index reflects prolonged intervals of high activity, interrupted only briefly by small decrements in synchronisation levels. This indicates that neurons are synchronised during the prolonged and accentuated bursting in GPi in the Parkinsonian state. As a result, the synaptic, inhibitory projection to the thalamus remains periodic, but predominantly strong, and is again only slightly reduced during short burst intervals, see Fig. 12b. The mean GPi synaptic activity (fluctuating around 0.7) is commensurate with this high synchronisation, which is supported by prolonged bursting of GPi neurons, only periodically interrupted by brief periods of reduced activity. The Fourier analysis reveals a dominant $\alpha$ /low$\beta$ activity with a strong peak at 10Hz, and a secondary at 18Hz, i.e. higher $\beta$ rhythm than the normal conditions. Importantly, the frequency spectrum of this bursting is much broader, as indicated by the corresponding linear approximation: The slope in Fig. 13b is increasing to $-2.5$ Hz/sec, which reflects that the higher frequencies contribute more (with respect to the normal case) to the power spectrum.

### Macroscopic dynamics during DBS

Simulating DBS, the macroscopic activities (as the order parameters *r* and *l* show) change dramatically (depending on a specific range of frequencies), introducing a state dissimilar to both normal or Parkinsonian states. The synchronisation index in Fig. 11c within the GPi shows a dynamic development over time, starting at values of $\approx$ 0.9, and then decreasing to $\approx$ 0.4, during the first 500 ms of tonic irregular firing in the GPi. After this period, the system adapts and oscillates at high frequencies with values depending on the applied frequency (e.g. between 0.8 and close to 1 in case of 184 Hz, between 0.6 and 0.8 in the case of 210 Hz). Similar dynamics emerge regarding the mean GPi synaptic activity, with a first transient period of 600 ms where the synaptic drive is decreased (to $\approx$ 0.2), and a second period ($t>600$ ms) where high synaptic activity is maintained (with fluctuations around a mean value which depend on the applied frequency (e.g. $\approx$ 0.6 in case of 184 Hz, $\approx$ 0.5 in case of 210 Hz) i.e. higher than under normal conditions and lower than under Parkinsonian. Surprisingly, this would appear to impose a strong inhibitory drive to the thalamus, see Fig. 12c. This, however, differs from the Parkinsonian condition as it is tonic high-frequency and not repeating bursting blocks appearing at $\beta$ frequency. This qualitative change in rhythmicity is crucial: While a relatively high inhibitory tonic drive seems to suggest strong thalamic inactivation, the contrary is the case. Due to the fact that the input to the thalamus is high-frequency tonic (peak power at 184 Hz), the GABAergic synapses are deactivated (see eqs. (4)). Indeed, the frequency analysis of the GPi activity shows a main peak seeming to resonate with the external DBS stimulus (around 60Hz) and the corresponding harmonics. Importantly, the peaks in the lower (normal state) and higher (Parkinsonian state) $\beta$ band disappear, while the slope changes back to a value around −3.1 Hz/sec, close to the normal case. Although this slope is close to normal conditions, it now reflects a narrow high-frequency band and not unclustered activity.

Taken together, the findings underscore the importance of synchronous regular and brief, clustered burst firing in the GPi for successful inhibition in the thalamus, which normally takes place at $\beta$ frequency. Although DBS thus does not restore normal rhythmicity in the GPi, the thalamic activity is disinhibited by the loss of synchronisation and clustering within the GPi.

## DBS efficiency depends on stimulation frequency

As the previous considerations show, the macroscopic order parameters *r* and *l*, which are defined in eq. (18) and (23), respectively, allow us to describe the effects of DBS stimulation and to compare the different states (normal, Parkinsonian, and DBS).

One critical parameter quantifying the effect of DBS simulation is the thalamic response with respect to the sensorimotor cortical signals. Faithful thalamic activation will send strong excitatory signals to cortex, see Fig. 1a, alleviating in this way the Parkinsonian symptoms (Guo et al. 2008). Naturally, under normal conditions the thalamic response should neither be completely uncoupled (with respect to the sensorimotor cortical signal), as under Parkinsonian conditions (see Fig. 7d), nor completely coupled as in an isolated cortico-thalamic system (Fig. 4). Indeed, under normal conditions, as shown in Fig. 6d, the thalamus follows the cortical input closely, but not in absolute synchrony.

In order to quantify the thalamic response to cortical input, under normal conditions or DBS with various stimulation frequencies, we define the response efficacy *R* of thalamic neurons as a mean value of the fraction of activated thalamic neurons (per stimulus) during simulated cortical activation. This simulation, as already discussed in Fig. 4, comprised sensorimotor current injections of length $\delta$ (5 ms) defined by the interval $[t,t+\delta ]$, with frequency of 40 Hz. The mathematical formulation of response efficacy *R* is thus defined as:

25 $$\begin{aligned} R(f)=\frac{1}{N_{\text {int}}}\sum \limits _{i=1}^{N_{\text {int}}} a_i(t), \end{aligned}$$

where $a_i(t)$ is the proportion of activated neurons, i.e. the number of activated neurons within the time interval of $[t,t+2\delta ]$ divided by the total number of thalamic neurons $N_{\text {THA}}=200$. The summation is taken over the number of intervals $N_{\text {int}}$, for times $500<t<1500$ ms, (i.e. $N_{\text {int}}=100$). Under normal conditions, as expected, *R* is approximately 0.5, i.e. suggesting a coupling to cortical input at a value offering the broadest dynamic range.

Under DBS at various frequencies, *R* shows a nonlinear behaviour, starting at 0 for low DBS frequencies (around 50 Hz) to values $>0$ with small peaks for frequencies around 70 and 130 Hz, see Fig 14. Very prominent peaks are found around 184 Hz, 210 Hz, and 244 Hz. Thus, there are three dominant optima at where the DBS maximises the thalamic response close to normal values of *R*. As Fig. 8 thus shows, the frequency of DBS is critical for thalamic firing outcome. At low frequencies (i.e. 50–150 Hz in Fig. 14), only few frequency bands can be found at which $R > 0$, while at this low range, the effects on thalamic activation are only transient, e.g. 50 Hz and 150 Hz (see Fig. 14). Exceptions are the small peaks at 70 and 130 Hz; Fig. 14; reasonably stable responses can thus be seen at 130 Hz. Only from 160 Hz onward, stable thalamic firing can be achieved by DBS. Interestingly, this is very similar to experimental findings in hemi-Parkinsonian rats: low-frequency DBS stimulation up to 75 Hz actually results only in transient effects, while permanent reductions of circling behaviour (the Parkinsonian equivalent in this animal model) were only achieved at DBS frequencies > 130 Hz (So et al. 2017).

Computing the Shannon entropy for the macroscopic variables *r*, *l* we confirm the optimal DBS frequency. As suggested by Dorval et al. (2008), Arle et al. (2018), Deco et al. (2012), an optimised DBS frequency can be achieved by regularisation of the whole basal ganglia activity (Dorval et al. 2008), i.e. minimisation of the entropy and thus a more ordered state. The Shannon entropy *E* is defined by

26 $$\begin{aligned} E_x(f)=-\sum _{i=0}^{N}P_f(x_i)\ln P_f(x_i), \end{aligned}$$

**Fig. 15:**
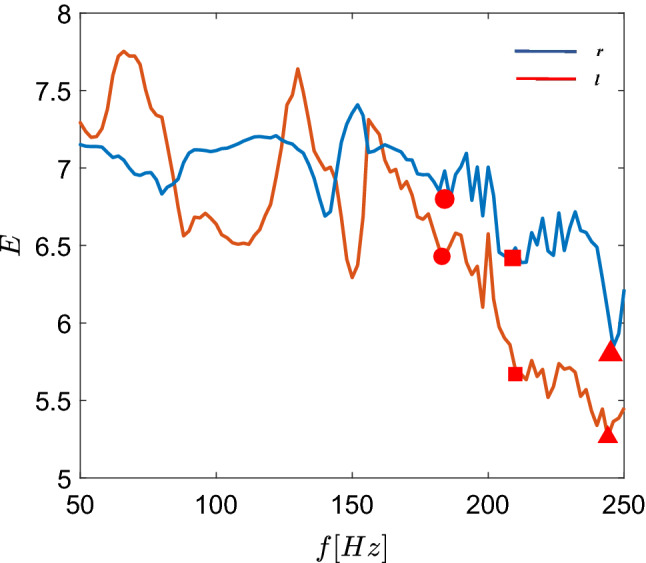
The entropy of the macroscopic variables (order parameters) *r*, *l*, as defined in equation (18) and (23). Computations were carried out for the time window [0, 1500] ms. The entropy decreases with increasing DBS frequency. The graph shows both single entropy values referring to (i) the synchronisation index (*blue line*) and to (ii) mean synaptic GPi activity index. The figure is in correspondence with response efficacy, see Fig. 14 with local minima at 184, 210, and 244 Hz marked with circle, square, and triangle, respectively

where $x_i$ expresses the macroscopic variable of interest (here $x=r, l$, as defined in equation (18) and (23)). The computation of the entropy with respect to the frequency was performed for the first 1500 ms including also the transient period of the first 500ms to be able to compare the initial response in experiments once the DBS is switched on (So et al. 2017; Mottaghi et al. 2020). The results are depicted in Fig. 15. From this figure it can be derived that the optimal frequencies for DBS are at 184 Hz and 210 Hz, where the entropy of the macroscopic variables (order parameters) *r*, *l* reach a local minima (in accordance with the thalamic response efficacy *R*, cf. Fig. 14, circle and square markers). A similar minima can be found when increasing *f* further at 244 Hz, which would require more energy for the DBS stimulation, likely raising the possibility of energy-dependent side effects.

## Discussion and conclusions

A large-scale basal ganglia computational model has been used to study network dynamics in movement disorders. The network model consists of 1700 interactive neurons with approximately 30000 connections (representing the *microscopic* level). The connectivity within the areas follows small-world topology where nearby neurons are densely connected; additionally, a small number of long-distance connections exist. The resulting structure combines properties of both regular and random topology, e.g. high clustering and at the same time short path length. Small-world structure enhances signal-propagation speed and synchronizability among the areas of the neuronal network.

For different values of parameters, the model reproduces phase transitions for normal, Parkinsonian, and DBS, in the emergent dynamics which are captured with suitable *macroscopic* order parameters for example the change from oscillatory to bursting behaviour of mean synaptic activity in Fig. 12a, b. Importantly, transitions produced by the model are consistent with the physiology and experimental observations of aberrant functionality of direct and indirect pathways.

Experimental findings of animal studies regarding the impact of STN-DBS on GPe, GPi, and thalamic firing could be approximated qualitatively. Specifically, in the transition from normal to Parkinsonian state shown in Figs. 6 and 7, the model alters the dynamics of GPe and GPi neurons due to varying levels of striatal inhibition. Remarkably, similar alternations in GPe/GPi dynamics were observed in Galvan and Wichmann (2008) (Fig. 2 therein) in monkeys and mice treated with MPTP (methyl-4-phenyl-1,2,3,6-tetrahydropyridine) (Galvan and Wichmann 2008; Ogawa et al. 1985). Specifically, in GPe single-cell recordings of monkey, the firing becomes sparser. This is indeed the case also in our model, see Fig. 7b. Concerning the GPi single cell activity, the firing rate increases strongly in the Parkinsonian state which is also reflected in our model, see Fig. 7c. In the STN area, the monkey single cell activity depicts bursting behaviour, while in our model burst firing only occurs sparsely, or not at all. However, the overall frequency increases similar to monkeys MPTP.

Furthermore, under DBS, in the model, the GPi and GPe fire tonically at high frequency, following STN firing at stimulation frequency (184 Hz), abolishing synchronised $\beta$ activity. This corresponds to the findings of McConnell et al. (2012), who showed that STN stimulation results in a sharp power peak at the same frequency also in GPe, and with findings of Wang et al. (2018) where DBS disrupts pallidal beta oscillations and the cortical coherence in Parkinson disease.

Regarding the $\beta$-band hypersynchrony hypothesis of Parkinson’s disease (Kühn et al. 2008), the model thus importantly shows that DBS actually abolishes $\beta$-band synchrony, which also in the model is prevalent in GP without stimulation. Hence, the model faithfully replicates what is known from clinical and animal model studies both on the cellular and on the network activity pattern levels. The model produces clusters of local travelling waves, more pronounced in the Parkinsonian state. Remarkably, in the experimental findings of Cagnan et al. (2015), it has been found that the analysis of local field potential recordings from the subthalamic nucleus and globus pallidus of patients with Parkinson’s disease shows beta-band propagation waves within the globus pallidus.

Further, the model predicts that a faster firing GPi projections under DBS will result in regular firing of the thalamus, which in turn will essentially follow the sensorimotor input—while under PD conditions, in fact thalamic firing was very sparse and irregular, and did not faithfully mirror cortical signals. These results are supported by studies in a Parkinsonian animal model in rhesus monkeys (Xu et al. 2008), where STN-DBS (as in Fig. 8) produces a change in the pattern and periodicity of neuronal activity in the basal ganglia thalamic network, resulting in a regular, higher-frequency firing pattern in the thalamus.

Moreover, macroscopic properties can be derived from our model. Since these essentially mirror local field potential activity, these properties can be used to test predictive modelling in future studies. For example, the model predicts a power law power spectrum, for the mean synaptic activity (see Fig. 13) with variable critical exponent *a*. Similar power law is reported in He et al. (2010) in the dynamics of visual cortex, hippocampus, and cerebellum with similar changes in the critical exponent *a*. They explained these differences due to the different areas of activation during the experiments. A power law behaviour (in basal ganglia areas and cortex) with deviations also is reported in West et al. (2018). In Huang et al. (2020) using local field potentials for 12 Parkinson’s disease patients, a power law activity in STN and cortex is reported. The STN activity shows strong variations of the exponent *a* during awake and loss of consciences state. In our case, this exponent is the slope of the linear approximation in Fig. 13. Our analysis shows that the slope can be distinguished between normal Parkinsonian and DBS state. Further, the model also allows to extract variables such as a macroscopic synchronisation and GPi synaptic activity indices which allow to predict the transitions during DBS application, which might pave the way for feedback control of DBS in the future (Popovych and Tass 2018, 2019; Manos et al. 2018).

Beyond this, the detailed analysis of the macroscopic parameters and derived values such as entropy also allow for an optimisation of DBS. As an outcome measure, the response efficacy of thalamic neurons reflects the degree of thalamic activity correlating to cortical input, which under normal conditions is $\approx$ 0.5. Critically, raising stimulation frequency beginning with 50 Hz, the first prominent entropy local minima of the two mesoscopic parameters precisely coincide with the first peak of the response efficacy *R* close to normal values ($\approx$ 0.23) at 184 Hz and more efficient frequencies over the 200Hz. These frequencies are not identical to the most commonly used in clinical settings i.e. 130 Hz, while in this frequency range, the model predicts sub-therapeutic action (see Fig. 14). In addition, one can argue that our modelling study does not fully replicate clinical findings, which show that at frequencies $>185$ Hz, side effects like dyskinesias seem to become more problematic (Dayal et al. 2017; Karl et al. 2019). Contrariwise, the match between the experimental findings in hemi-Parkinsonian rats (So et al. 2012, 2017; Mottaghi et al. 2020) and the response efficacy *R* is very interesting. In So et al. (2012) the authors describe a sustained depression of circling to values smaller than 5 turns per minute only at frequencies > 75 Hz. This incidentally matches well with our findings, which show peaks in the response efficacy *R* at  70 Hz and 130 Hz, and then at peaks of  180, 210, and 245 Hz, in close approximation to the effective frequencies reported in So et al. (2012, 2017), i.e. 185 and 260 Hz. Remarkably, similar frequency-dependent behaviour is reported in the recent study of Mottaghi et al. (2020), where strong impact of stimulation frequency on the induced rotation (caused by DBS) was found in the range of 250Hz.

Our mathematical model extends previous computational work (So et al. 2012; Dorval et al. 2010) by using a larger number of neurons for each basal ganglia areas and complex small-world connectivity. Additionally, we propose different approximations in the DBS frequency. The frequency analysis of Dorval et al. (2010) differs from our investigation since they focus on perturbations around 130 Hz (Fig. 1 and 2 in Dorval et al. 2010). In So et al. (2012) the activation patterns of local cells and fibres passage are studied with respect to the fidelity of thalamus. Our model suggests that the basal ganglia network behaviour to DBS stimulates frequency has a strong nonlinear response similar to resonating mechanical systems with optimal frequencies above 130Hz, suggesting the investigation of DBS treatment beyond the 130Hz. In summary, the mathematical model and the analysis is considered a powerful tool to explore the effects of parameter-dependent changes of DBS and to optimise the medical treatment.

## Outlook

One major future topic will be the study of network topological variations and the impact on the emergent dynamics. Thus, the functional effects of neuroanatomical changes observed in Parkinson’s disease (Prakash et al. 2016), such as massive decreases of dendritic length of medium spiny neurons (Stephens et al. 2005), could be modelled. Considering movement disorders beyond Parkinson’s disease, the model could also be extended to further elements of the basal ganglia, to gauge the effect of alterations in cortico-striatal communication such as those observed in dystonic hamsters (Köhling et al. 2004), where alternations in excitability in dystonic tissue were described to be related to both changes in intrinsic neuronal properties and presynaptic release probability at glutamatergic synapses. Furthermore, our analysis can be extended beyond the initial interval of the first 1.5 sec (which contains also transient effects). The analysis of the long-time behaviour under DBS including the effects of neuromodulators and the changes in synapses functionality (network plasticity) (Marschler et al. 2014; Morrison et al. 2008; Droste et al. 2013), is an interesting subject for future studies.

In future work, it will be important also to investigate the parameter dependence of network dynamics, using numerical bifurcation tools for multiscale problems (Spiliotis and Siettos 2011; Marschler et al. 2014; Moon et al. 2015; Schmidt et al. 2018), for an in-depth understanding of the functional network changes occurring in movement disorders.
